# Left-sided Macro-reentry or Right-sided Focal Tachycardia in Patients with Prior Pulmonary Vein Isolation: A Tale of Two Atria

**DOI:** 10.19102/icrm.2021.121104

**Published:** 2021-11-15

**Authors:** Khalil Kanjwal, Asim Kichloo, Khalid Mohiuddin Mir, Abdul Qadir Haji

**Affiliations:** ^1^Department of Electrophysiology, McLaren Greater Lansing Hospital, Lansing, MI, USA; ^2^Department of Internal Medicine, Samaritan Medical Center, Watertown, NY, USA; ^3^Department of Internal Medicine, Central Michigan University, Saginaw, MI, USA; ^4^Super Specialty Hospital, Government Medical College, Srinagar, Kashmir, India; ^5^Department of Cardiology, Martinsburg VA Medical Center, Martinsburg, WV, USA

**Keywords:** Atrial fibrillation, atrial flutter, atrial tachycardia, left atrium, pulmonary vein isolation

## Abstract

We report on three patients with prior pulmonary vein isolation who presented with atrial tachycardia/atrial flutter. During electrophysiology study, the whole tachycardia cycle length was mapped to the left atrium. Multiple ablation attempts failed to terminate the tachycardia and, subsequently, right atrial mapping revealed a focal early site of origin near the superior vena cava–right atrial junction in two patients and outside the coronary sinus ostium in one patient. In this report, we discuss the probable mechanism of these tachycardias.

## Introduction

Pulmonary vein isolation (PVI) has emerged as the most effective rhythm-control strategy. The number of atrial fibrillation (AF) ablation procedures has increased and so has the number of patients developing post-PVI atypical atrial flutter (AFL) and atrial tachycardia (AT). Herein, we report three patients who developed post-PVI AT/AFL. The whole tachycardia cycle length (TCL) was mapped to the left atrium (LA), and a macro-reentry type of activation and propagation was noted in the LA. All these patients had an unsuccessful ablation from the LA. Subsequently, the right atrium (RA) was mapped. The tachycardia was found to have a focal origin near the superior vena cava (SVC)–RA junction in two patients and just beneath the coronary sinus (CS) ostium in another. In this report, we present the probable mechanism of these observations. Verbal informed consent was obtained from the patients for their anonymized information.

## Case presentations

### Case 1

**Figure 1:**
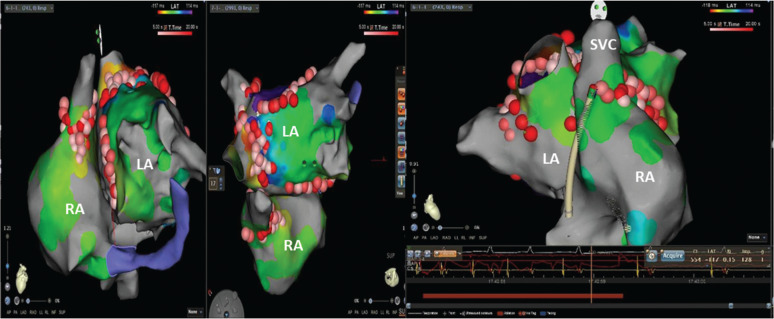
The activation and propagation were suggestive of an LA reentry; however, a focal ablation in the SVC area resulted in the termination of the tachycardia. LA: left atrium; RA: right atrium; SVC: superior vena cava.

A 70-year-old woman with a history of persistent AF underwent PVI. Unfortunately, she developed post-PVI AFL. The patient came to the electrophysiology (EP) lab with a TCL of 240 ms. The activation map revealed a reentry-appearing tachycardia, and the whole TCL was mapped to the LA. The propagation and ripple maps revealed activation originating from the anterior right superior pulmonary vein (RSPV). There was some gap in the right-sided veins, and they were re-isolated. Multiple attempts at ablation in the LA near the RSPV failed to terminate the tachycardia. At that point, it was decided to map the RA. The activation map revealed an early appearing focal area in the SVC adjacent to the area in the anterior RSPV. It was decided to isolate the SVC, and as the ablation was continued on the septal side of the SVC, the tachycardia terminated **(Figure 1)**.

### Case 2

**Figure 2:**
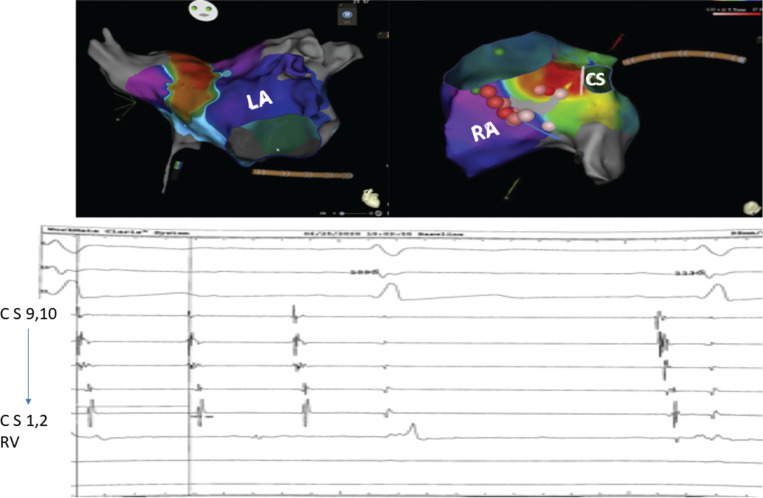
Activation and propagation in the LA boast a reentry appearance. Activation and propagation maps in the RA show a focal mechanism with early activation near the CS ostium. Ablation in this area resulted in the termination of the tachycardia (electrogram). CS: coronary sinus; LA: left atrium; RA: right atrium.

A 68-year-old man with a history of persistent AF developed post-PVI AT/AFl. The patient was taken to the EP lab and was found to be in AT/AFL with a TCL of 340 ms and proximal to distal CS activation. The whole LA was extensively mapped using the CARTO^®^ mapping system (Biosense Webster, Diamond Bar, CA, USA). The pulmonary veins were found to be isolated. The activation pattern revealed an area of early activation noted just superior to the RSPV. The activation map captured the whole TCL from the LA. The propagation map was suggestive of a reentry mechanism. However, multiple attempts at ablation in the left chamber failed to terminate the tachycardia. Subsequently, the RA was mapped, and the activation map revealed an early site of activation just outside the CS ostium **(Figure 2)**. Ablation in this area terminated the tachycardia **(Figure 2)**. Ablation was subsequently performed in the cavotricuspid isthmus as well.

### Case 3

**Figure 3:**
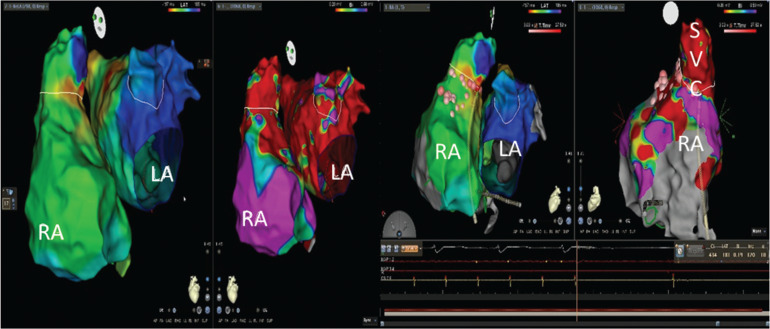
Activation map of a post-PVI AFL. The whole TCL was mapped from the LA. Propagation was consistent with the macro-reentry in the LA. However, the tachycardia was terminated by a focal ablation in the adjacent SVC–RA junction. LA: left atrium; RA: right atrium.

A 73-year-old man with a history of symptomatic persistent AF, coronary artery bypass graft, hypertension, and diabetes mellitus who had previously undergone AF radiofrequency (RF) ablation with PVI and roof, mitral isthmus, and cavotricuspid isthmus ablation presented with AFL with proximal to distal activation in the CS. Given his prior extensive LA ablation, it was decided to map the LA first. After transseptal access, extensive fast anatomical mapping, voltage mapping, and activation mapping were performed. All veins were isolated. Activation mapping revealed that the whole TCL was mapped in the LA. Also, there was an area in the roof near the RSPV anteriorly that showed a gap in the previous roof ablation line **(Figure 3)**. Entrainment in this area resulted in a close post-pacing interval (PPI) suggestive of the area being part of the circuit. Entrainment also revealed that the cavotricuspid isthmus and the lateral RA were not part of the circuit. Ablation performed in this area prolonged the TCL without terminating the tachycardia, and no change in activation was noted. Subsequently, we decided to map the RA, and activation mapping, fast anatomical mapping, and voltage mapping of the RA were performed. There was an area of early activation near the junction of the SVC and the RA, and heterogenous scarring was noted in this area on the voltage map. This area was anteriorly adjacent to the RSPV roof. Entrainment from this area also resulted in a close PPI again, suggestive of the area being part of the circuit. Ablation was finally performed near the RA–SVC junction, resulting in TCL prolongation and tachycardia termination **(Figure 3)**.

## Discussion

PVI is a commonly performed procedure for the management of AF. Some of these patients develop post-PVI atrial tachyarrhythmias. The mechanism of these tachycardias can be either focal or macro-reentrant. These post-PVI tachyarrhythmias commonly arise in the LA and are thought to occur because of the incomplete isolation of pulmonary veins and gaps in linear LA ablations.^1^ There were a few interesting observations noted in our series, which are discussed below.

### The whole tachycardia cycle length was mapped to the left atrium

During activation and propagation mapping, all patients demonstrated a reentry-appearing activation and propagation. The whole TCL was also mapped to the LA, thus making it likely a left-sided macro-reentrant tachycardia. Thus, one of the things this series highlights is that activation and propagation maps alone cannot always be reliable, especially in patients who had undergone prior PVI and linear ablations. These patients have complex atrial scars and slow conduction zones that can result in a reentry-appearing activation and propagation mechanism. Based on the observation that the whole TCL was mapped in the LA, it was presumed that the tachycardia originated in the LA. However, multiple attempts at ablation in the LA failed to terminate the tachycardia.

### Termination of the tachycardia from the right side

After the tachycardia could not be terminated from the left side, it was decided to map the contralateral chamber. In the RA, the tachycardia was localized and successfully terminated with a focal ablation at the RA–SVC junction in two patients and outside the CS ostium in one patient. Termination of the tachycardia proved that it was originating from the RA. Focal AT is characterized by atrial activation starting rhythmically at a small area (focus) from where it spreads centrifugally.^2^ There is generally an electrically silent period in the atrial cycle length that, in the electrocardiogram, is reflected by an isoelectric line between atrial deflections. Intracardiac mapping will show significant portions of the cycle length without recorded activity, even when recording from the entire RA, LA, and/or CS **(Figure 3)**. However, in the presence of complex intra-atrial conduction disturbances, intra-atrial activation may extend over a large portion of the cycle length, and conduction spread may follow circular patterns suggestive of macro-reentrant activation on these propagation and activation maps. This is particularly true in the post-PVI era where complex lesion sets involving the LA create conduction barriers and zones of slow conduction promoting macro-reentry. In one study, the overall incidence of post-PVI AFL after the blanking period was 4.5%, with only 1.6% of all patients developing confirmed cavotricuspid isthmus-dependent AFL.^3^ Three-dimensional electroanatomic mapping has vastly expanded the real-time visualization of this macro-reentry, usually characterized as meandering wavefronts within the scarred atria.^4^

Bi-atrial tachycardia, although a rare form of macro-reentrant tachycardia, is increasingly being recognized and is usually the result of either previous ablation, surgery, or intrinsic disease/scarring along the septum.^5^ Ip et al. elegantly outlined the differential diagnosis of such patients with entrainment from the low lateral RA, proximal CS, and distal CS with a PPI within 30 ms of TCL. The differential diagnosis included (1) single-loop macro-reentry involving the RA and LA; (2) double-loop reentry in a figure-of-eight pattern around the tricuspid and mitral valves; and (3) RA flutter with the LA participating as a passive bystander during entrainment. In our series of three patients, during activation and propagation mapping, all patients demonstrated mapping apparently consistent with the LA macro-reentry. The whole TCL was mapped to the LA, thus giving the appearance of a left-sided macro-reentrant tachycardia. However, the unique feature in all our patients was that the tachycardia terminated with a focal ablation from a discrete RA site that was either a part of or feeding directly into a seemingly macro-reentrant circuit involving the LA. We propose that the LA was, in fact, passively activated from the RA tachycardia and was masquerading as an LA macro-reentry. This would be characterized as showing bi-atrial dependence rather than the true bi-atrial tachycardia propagation.

One would argue that we mapped the wrong chamber to begin with. However, it is not uncommon that most post-PVI flutters/tachycardia originate from the LA. In the real world, one tends to map the LA if the patient presents with post-PVI tachycardias. In our patients, what led to the ablation attempts in the LA was not only the activation/propagation maps but also the fact that the whole TCL could be mapped to the LA. From this series, we learn that one should not rely on the propagation characteristics in a scarred atrial chamber and having a low threshold of mapping the RA, even when the activation map suggests reentry in the LA. One should analyze the overall activation pattern in both atria rather than the intra-atrial activation that may extend over a large proportion of the cycle length in one chamber. Mapping the RA chamber in an apparently left-sided–looking macro-reentrant flutter in our series led to the successful ablation of these tachycardias.

In patients with a redo RF ablation, the presence of scar from the previous ablation can alter the lesion formation by insulating and redirecting the heat propagation from the ablation source. This may eventually render the ablation procedure ineffective in patients with extensive scarring.^6^

### The successful site of ablation in the right atrium was adjacent to the left atrium

The other interesting observation was that the successful site of ablation was adjacent to the LA. Thus, in post-PVI AFL patients, in addition to LA mapping, an extensive mapping in the adjacent SVC, septum, and near the CS ostium should be routinely performed. Although not truly a bi-atrial tachycardia, we believe that our series showed a degree of bi-atrial dependence. Because of the anatomical proximity, the AT arising in these areas may directly feed into the LA and may seemingly appear as an LA macro-entry. When a macro-reentrant LA tachycardia is mapped in the RA chamber, it usually appears as a focal tachycardia with the earliest activation seen in the septum. In a similar fashion, we believe that a focal tachycardia near the septum, SVC, and CS ostium may appear as a macro-reentry when mapped in the LA, especially in patients with extensive scarring in the LA.

We also noted a diffuse area of early activation in the septum near the RSPV in all these patients. We use a term “septal blush” on the LA activation map, which may suggest a possibility of successful ablation from the RA when ablation from LA sites is unsuccessful.

## Conclusion

In post-PVI atrial arrhythmia, due to complex LA scarring and conduction abnormalities, a focal RA tachycardia can appear as a left-sided macro-reentry tachycardia. Careful mapping in the adjacent sites, including the SVC, septum, and CS ostium, can result in the successful identification of the successful target site for the ablation.

## References

[1] Chen S-A, Chiang C-E, Yang C-J (1994). Sustained atrial tachycardia in adult patients. Electrophysiological characteristics, pharmacological response, possible mechanisms, and effects of radiofrequency ablation. Circulation.

[2] Saoudi N, Cosío F, Waldo A (2001). A classification of atrial flutter and regular atrial tachycardia according to electrophysiological mechanisms and anatomical basis. A Statement from a Joint Expert Group from the Working Group of Arrhythmias of the European Society of Cardiology and the North American Society of Pacing and Electrophysiology. Eur Heart J.

[3] Baman JR, Kaplan RM, Diaz CL (2020). Characterization of atrial flutter after pulmonary vein isolation by cryoballoon ablation. J Interv Card Electrophysiol.

[4] Asferg C, Chen X, Pehrson S, Jacobsen PK (2019). Catheter ablation of atypical flutter using new 3-dimensional electroanatomic mapping software focusing on areas of conduction block. Heart Rhythm Case Rep.

[5] Ip JE, Cheung JW, Liu CF, Thomas G, Markowitz SM, Lerman BB (2016). Biatrial tachycardia: distinguishing between active and passive activation. Circ Arrhythm Electrophysiol.

[6] Tao S, Guttman MA, Fink S (2019). Ablation lesion characterization in scarred substrate assessed using cardiac magnetic resonance. JACC Clin Electrophysiol.

